# Adjunctive Application of Hyaluronic Acid in Combination with a Sodium Hypochlorite Gel for Non-Surgical Treatment of Residual Pockets Reduces the Need for Periodontal Surgery—Retrospective Analysis of a Clinical Case Series

**DOI:** 10.3390/ma15196508

**Published:** 2022-09-20

**Authors:** Daniel Diehl, Anton Friedmann, Pheline Liedloff, Rico Marvin Jung, Anton Sculean, Hakan Bilhan

**Affiliations:** 1 Department of Periodontology, Faculty of Health, Witten/Herdecke University, 58455 Witten, Germany; 2 Institute of Pharmacology and Toxicology, Faculty of Health, Witten/Herdecke University, 58455 Witten, Germany; 3 Department of Periodontology, School of Dental Medicine, University of Bern, 3012 Bern, Switzerland

**Keywords:** hyaluronic acid, sodium hypochlorite, periodontitis, non-surgical periodontal therapy

## Abstract

The comprehensive treatment of periodontitis stage 2 to 4 aims at the resolution of periodontal inflammation and “pocket closure”, which implies a residual probing depth of ≤4 mm and a negative BoP. However, supportive periodontal therapy (SPT) regularly leaves behind persistent periodontal pockets with 5 or more mm in residual PPD and sites that often re-colonize and re-infect. Various adjunctive options for subgingival instrumentation have been proposed to enhance the antimicrobial effects to better control the re-infection of these residual sites. The locally applied adjuncts, based on their anti-inflammatory effect, are sodium hypochlorite antiseptic cleaning gel and cross-linked hyaluronic acid (xHyA). Both recently moved into the focus of clinical research on non-surgical and surgical therapy for periodontitis. The surgical use of xHyA indicates regenerative potential, supporting periodontal regeneration. This case series retrospectively analyzes the clinical benefits of the consecutive flapless application of sodium-hypochlorite-based cleaning gel and xHyA at the SPT to achieve pocket closure, thereby reducing the need for periodontal surgery. In 29 patients, 111 sites received the treatment sequence. At 6-month re-evaluation, an overall PPD reduction exceeding 2 mm was achieved, associated with a similar CAL gain (2.02 mm); the bleeding tendency (BoP) was reduced by >60%. Pocket closure occurred in almost 25% of all the sites. Within their limits, the present data suggest that the proposed combined adjunctive treatment of residual active periodontal sites yielded significant improvement in the clinical parameters. Further studies in RCT format are required to confirm these observations.

## 1. Introduction

Non-surgical periodontal treatment (NSPT) results in improved probing depth, clinical attachment level, and bleeding tendency [1]. The purpose of NSPT is the resolution of periodontal inflammation and a reduction in pocket-probing depth (PPD) to 4 mm or less, resulting in pocket closure. However, residual or recurring pockets exhibiting PPD values ≥4 mm are regularly found at re-evaluation. Residual periodontal pockets facilitate the accumulation of biofilm, leading to dysbiosis within the re-colonized subgingival habitat and, thus, to persistent inflammation [2,3]. Moreover, long-term data confirm the association between residual PPD and increased risk of tooth loss [4]. Therefore, as recommended in the European Federation of Periodontology (EFP) guidelines, continuous supportive periodontal therapy (SPT) accompanied by repeated instrumentation is imperative for sustained periodontal stability [5].

In an effort to improve the outcome of non-surgical instrumentation, a variety of adjunct treatment modalities are used. In addition to systemic antibiotics, a plethora of locally administered adjunctives seek to minimize both PPD and bleeding tendency, thereby facilitating the closure of the periodontal pocket.

Most of these adjunctive treatments are based upon the antimicrobial effects delivered by either photodynamic therapy (PDT) or the use of local antibiotic chemotherapy, preferably applied as a device with sustained release kinetics [6,7,8,9,10]. Furthermore, gelatin chips sustainably releasing chlorhexidine have been described [11,12,13]. Addressing the limitations of subgingival instrumentation on pocket-closure frequency, a recent systematic review and meta-analysis evaluated the additional benefit of locally applied adjunctive therapies. Even though the authors found effects of statistical significance, the magnitude of these benefits was deduced to be rather irrelevant to clinical success in terms of pocket closure [14]. Furthermore, the microbiological analysis of samples retrieved from persistent deep pockets before and after repeated local metronidazole application revealed high counts of periodontal pathogens [9].

By contrast, a novel amino-acid-buffered sodium hypochlorite cleaning gel exhibiting antimicrobial potential was significantly effective in improving the outcome of non-surgical therapy and, thus, significantly reduced counts of Gram-negative pathogens in an artificial biofilm model [15,16]. 

Another strategy to improve periodontal parameters is the local administration of regenerative biologics. In an attempt to harness its well-documented regenerative properties, a recent multi-center randomized controlled trial investigated the effect of enamel-matrix derivatives (EMDs) as an adjunct to the NSPT of patients situated in SPT [17]. The authors were able to show significantly greater pocket closure for sites treated with adjunctive EMD, demonstrating biologics-based regenerative technologies as promising supplements for non-surgical therapy. 

Furthermore, a review with a meta-analysis showed that the adjuvant non-surgical administration of hyaluronic acid (HA) resulted in an improvement in both clinical attachment and probing depth [18]. Currently, however, there is a lack in protocols for adjuncts to NSPT combining both antimicrobial and regenerative properties. In this retrospective case series, we propose a novel two-step approach consisting of an amino-acid-buffered sodium hypochlorite cleaning gel to assist in the decontamination of the root surface, followed by the concomitant application of a cross-linked hyaluronic acid gel (xHyA) to facilitate healing and, thus, pocket closure. We report the retrospective analysis of 6-month clinical follow-up data from patients who qualified for this therapy.

## 2. Materials and Methods

The local ethics committee at the Witten/Herdecke University approved this retrospective evaluation of a clinical case series (S-203/2021). All the analyzed cases had been diagnosed with stage 2 to 4 periodontitis previously and had already undergone comprehensive periodontal therapy, as proposed by the EFP guidelines [5,19]. Four calibrated specialists and residents at the Department of Periodontology of Witten/Herdecke University were responsible for all treatment steps. Calibration of investigators was evaluated by analysis of variance (ANOVA), followed by Tukey’s post hoc analysis for multiple comparisons (*p* > 0.59 for all investigators). The decision to administer systemic antibiotics strictly complied with the EFP guidelines, following completion of initial subgingival instrumentation.

### 2.1. Inclusion Criteria

The proposed treatment applied to sites that exhibited persistent deep pocket depths after patients had undergone consecutive SPT re-evaluations at least twice. Sites ascribed to the treatment by protocol had never been subjected to any surgical intervention, even though patients may have received periodontal surgery at other sites. Specifically, persistent and recurrent periodontal pockets displaying ≥5 mm in PPD with positive BoP were included. The number of sites per patient assigned to the therapy was unrestricted. There was no limit to the localization of residual or recurrent pockets, and single- and multi-rooted teeth were included. In teeth with high PPD associated with furcation involvement of more than Class 1, only the change in vertical component of the defect was analyzed for this report.

### 2.2. Treatment Sequence

**Figure 1:**
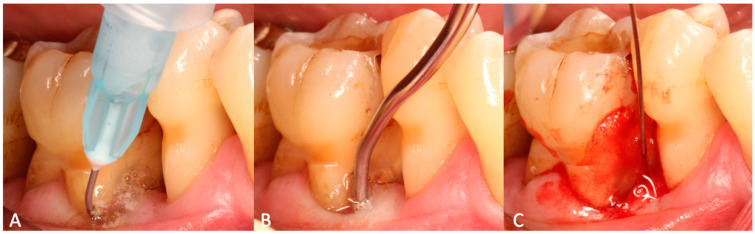
Visualization of the applied treatment protocol. (**A**) Application of chloramine gel to the pocket for 30–45 s. (**B**) Scaling and root planning is performed. Chloramine gel may be applied repeatedly until non-surgical treatment is deemed sufficient. (**C**) Cross-linked hyaluronic acid (xHya) is applied to the pocket until plenished.

Four calibrated operators treated all patients; the operators agreed upon the treatment protocol before the first application. Following supragingival mechanical instrumentation, each site received subgingivally administrated sodium hypochlorite cleaning gel (Perisolv; Regedent AG, Zürich, Switzerland) for 30 to 45 s to support chemical disinfection and improve the scaling outcome. Subgingival instrumentation was carried out with Gracey curettes (Deppeler, American Dental Systems, Munich, Germany). The sodium hypochlorite cleaning-gel application was repeated until the instrumentation was considered sufficient (Figure 1). Sufficient instrumentation was attained when root surfaces exhibited smooth surfaces upon probing with an explorer probe (ODU 11/12 DH2, Deppeler, Rolle, Switzerland). Subsequently, 0.3 mL of the cross-linked hyaluronic acid gel (xHyA; hyaDENT BG, Regedent AG, Zürich, Switzerland) was applied into the subgingival pocket in a flapless manner until plenished. Patients were instructed to uphold daily mechanical biofilm control by means of interdental brushes and a toothbrush. Measures for oral hygiene were not adjusted in the operated area. Neither systemic antibiotics nor antiseptics for rinsing were prescribed by protocol. Within the next 7 days, a repeated subgingival xHyA application (0.3 mL) was conducted combined with the oral hygiene control. The first re-evaluation took place 5–6 months after treatment and the subsequent SPT interval was set to 3 months for a 12-month period. At the 12-month re-evaluation, a periapical radiograph taken with the parallel technique was obtained to verify the crestal bone level.

### 2.3. Statistical Analysis

For all obtained datasets, a descriptive data analysis was performed. Further statistical analyses included the Shapiro–Wilk, Kolmogorov–Smirnov, and D’Agostino–Pearson tests to assess data distribution. CAL gain and PPD reduction (pre–post) were both calculated by Wilcoxon signed-rank test, respectively. *p*-values of ≤0.05 were considered significant.

## 3. Results

This retrospective analysis included 29 patients with 111 treated teeth/sites, ranging from 1 to 17 per patient. The mean age was 54.6 years, and 69% were female (20:9; 69% vs. 31%). All patients were normo-glycemic and 7% (*n* = 2) were smokers. Table 1 discloses the demographics, habits, and health condition of the participants. All of them participated in the SPT program offered by the Department of Periodontology.

**Figure 2:**
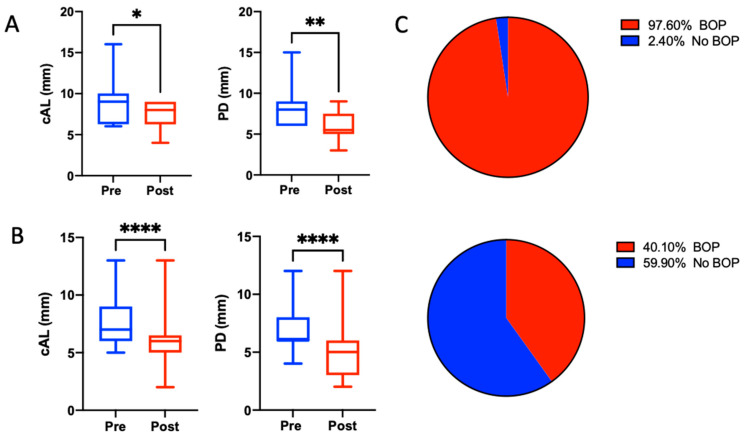
Boxplots for clinical parameters before and after the treatment sequence of non-furcation-involved (**A**) and furcation-involved (**B**) sites. Whiskers represent minimum and maximum values. * *p* < 0.05, ** *p* < 0.01, **** *p* < 0.0001. (**C**) Amount of sites exhibiting bleeding on probing before (upper) and after (lower) the treatment.

The mean PPD at baseline was 7.19 (±1.89) mm, and the CAL loss was 7.96 (±2.2) mm; 97.6% of all sites presented with positive BoP. Consecutive six-month re-evaluation revealed an overall PPD reduction of 2.04 mm and a clinical attachment level gain of 2.02 mm, indicating that no further progression in gingival recession occurred. The BoP frequency decreased to 40.1%. Stratified by furcation involvement (12 teeth), the mean CAL gain was 1.5 mm (*p* = 0.0195), whereas the treatment of single-rooted teeth resulted in a 2.04 mm (*p* < 0.001) CAL gain (Table 2 and Table 3, Figure 2). Both measurements yielded statistically significant differences compared to the baseline values. In terms of pocket closure, 25 out of 99 (25.25%) sites in the single-rooted teeth exhibited pocket closure, with a PPD < 4 mm and a negative BoP.

## 4. Discussion

This retrospective case series shows that the combination of an antiseptic adjunctive cleaning gel and xHyA applied subgingivally for the treatment of persistently deep periodontal pockets at SPT visit yielded clinically relevant improvements in PPD reduction, CAL gain, and BoP frequency. The follow-up of the reported cases revealed statistically significant improvement in all three of these parameters. The overall CAL gain exceeded 2 mm on average in sites previously classified as non-responding and persistent. Although a minor number of treated sites exhibited complete pocket closure after three to six months, the two-component flapless adjunctive treatment considerably reduced the need for periodontal surgery. Sites ascribed to surgical step3 therapy according to the EFP guidelines clinically improved to such an extent that the periodontal surgery became redundant. To the best of our knowledge, this is the first report of the combined use of antiseptic and biologic approaches in flapless periodontal treatment. As each site received both adjunctive materials administered at one visit, we must emphasize that a discussion of the individual contributions to the results appeared unnecessary.

Recent in vitro, pre-clinical, and clinical studies investigated either the sodium hypochlorite cleaning gel or the xHyA application in a separate manner. The antimicrobial effects of the sodium hypochlorite cleaning gel became evident [16,20]. Cell-based experiments also disclosed the high level of cytocompatibility of its compounds [20,21]. However, the benefits of adjunctive sodium hypochlorite cleaning gel for NSPT remain controversial. Sodium hypochlorite gel failed to affect the clinical outcome of ultrasonic or manual subgingival instrumentation in SPT treatment. Nevertheless, its use was associated with significantly reduced recolonization of the sites by *T. denticola* and *T. forsythia* [22]. By contrast, the adjunctive benefit of sodium hypochlorite gel formulation for minimally invasive non-surgical therapy (MINST) was positively evaluated by a recent RCT [15]. The authors compared the outcome of step-2 therapy after delivering it to untreated stage-3 and -4 periodontitis patients in both study arms. Moreover, in an RCT study from a Scandinavian research group, diabetic foot ulcers resolved significantly quicker under treatment with this cleaning gel formulation than those in the control group [23].

Hyaluronic acid (HA) is a glycosaminoglycan heteropolysaccharide and, in its native form, it is both a light-molecular-weight (LMWHA) and a high-molecular-weight long polymer (HMWHA) [24]. HA is an important natural component of the extracellular matrix and is almost ubiquitously present in mammalian tissues, including the periodontium [25]. Several studies confirmed bacteriostatic [26,27], fungostatic [28], anti-inflammatory [29], anti-edematous [30], osteoinductive [29,31,32,33], and pro-angiogenic [34] properties of HA. In animal studies on skin wounds, HA promoted enhanced connective-tissue elasticity and healing, improved re-epithelialization, and appeared to increase microvascular density [34,35]. HA sufficiently improved wound healing in extraoral wounds, skin ulcers, and intraoral injuries [36,37,38].

The potential of xHyA to promote periodontal regeneration became a subject in a recent series of histological evaluations in dogs’ mandibles. The histomorphometric assessments revealed that xHyA-treated intraosseous and furcation sites formed significantly greater areas of new cementum and periodontal ligament fibers on previously exposed root surfaces. Similar observations were made from the same treatment sequence applied in gingival recessions [39,40,41].

The clinical results mediated by xHyA indicated a substantial benefit, which was corroborated by both a recent RCT study and a case series [42,43]. Beyond the positive effects of xHyA unfolded in the surgical context, its adjunctive use in NSPT yielded inconsistent outcomes in clinical studies [44,45,46].

In our retrospective analysis, we found a significant probing-depth reduction accompanied by a significant gain in clinical attachment (Figure 2). Moreover, the needlessness of root conditioning and drying the wound area increased the ease of handling and delivered strong arguments in favor of xHyA as an adjunct to flapless subgingival instrumentation, as well as accounting for its hygroscopic/wound-stabilizing and regenerative properties. In addition, compliance with the second visit scheduled for repeated xHyA application was high in all the patients. With respect to the proposed protocol, the sodium hypochlorite cleaning-gel application may offer further advantages to NSPT by means of improving the mechanical biofilm removal, thus enhancing the effects of the xHyA. Therefore, we consider the proposed protocol highly beneficial for NSPT. However, the presented results require further confirmation by randomized controlled clinical trials, which may also account for the exposure time and application frequency of the hypochlorite gel.

## Figures and Tables

**Table 1:** Patient demographics and mean clinical parameters before (pre) and after (post = 6 months) the treatment. CAL = clinical attachment level, PPD = probing pocket depth, BOP = bleeding on probing, * = Wilcoxon signed-rank test.

Patients (Sites)	29 (111)			
Mean Age (Range)	54.6 (39–75)			
Sex				
− Male (%)	9 (31%)			
− Female (%)	20 (69%)			
Smokers (%)	2 (7%)			
Diabetes (%)	0			
		Pre	Post	CAL gain/PPD Reduction
CAL	Mean (SD)	7.96 (±2.2)	5.95 (±1.8)	+2.02 mm (*p* < 0.0001) *
	Median	7	6	
	Min	2	2	
	Max	9	13	
PPD	Mean (SD)	7.19 (±1.89)	5.16 (±1.81)	−2.04 mm (*p* < 0.0001) *
	Median	6	5	
	Min	4	2	
	Max	15	12	
BOP		97.6%	40.1%	

**Table 2:** Descriptive statistics of PPD and CAL development in furcation-involved sites after combined chloramine and xHya treatment. Pre = baseline, Post = 6 months post treatment, * = Wilcoxon signed-rank test.

Furcation Involved (n = 12)		Pre	Post	CAL Gain/PPD Reduction
CAL	Mean (SD)	9.08 (±2.88)	7.58 (±1.73)	+1.50 mm (*p* = 0.0195) *
	Median	9	8	
	Min	6	4	
	Max	16	9	
PPD	Mean (SD)	8.25 (±2.59)	5.833 (±1.75)	−2.42 mm (*p* = 0.002) *
	Median	8	5.5	
	Min	6	3	
	Max	15	9	

**Table 3:** Descriptive statistics of PPD and CAL development in sites without furcation involvement after combined chloramine and xHya treatment. Pre = baseline, Post = 6 months post treatment, * = Wilcoxon signed-rank test.

No Furcation Involved (n = 99)		Pre	Post	CAL Gain/PPD Reduction
CAL	Mean (SD)	7.93 (±2.03)	5.89 (±1.87)	+2.04 mm (*p <* 0.0001) *
	Median	7	6	
	Min	5	2	
	Max	13	13	
PPD	Mean (SD)	6.96 (±1.68)	5.15 (±1.86)	−1.81 mm (*p* < 0.0001) *
	Median	6	5	
	Min	4	2	
	Max	12	12	

## Data Availability

The data presented in this study are available on request from the corresponding authors. The data are not publicly available because they were derived from patients.
